# Supplementary material for: The tricellular vertex-specific adhesion molecule Sidekick facilitates polarised cell intercalation during Drosophila axis extension
Source: PLoS Biol. 2019 Dec 5;17(12):e3000522. doi: 10.1371/journal.pbio.3000522 (PMC6894751; doi:10.1371/journal.pbio.3000522)
Supplement: S1 Text — GBE, germband extension; Sdk, Sidekick. (DOCX) [file pbio.3000522.s006.docx]

**Supplementary Methods: Vertex model of *Drosophila* germband extension in wild-type and *sdk* mutant**

We use mathematical modelling to investigate the mechanical implications of actomyosin planar polarisation during *Drosophila* germband extension in a *sidekick* mutant, modifying a recent vertex model of wild-type behaviour (Tetley et al., 2016). Vertex models describe epithelial mechanics by considering the polygonal tessellation that cells’ adherens junctions form in two dimensions (Farhadifar et al., 2007; Fletcher et al., 2014). In such models, the movement of junctional vertices and the intercalation of cells are governed by elastic parameters modulating the line tension along cell cortices, the stiffness of the cell cortex and cell bulk stiffness.

In a recent model of an actively intercalating tissue (Tetley et al., 2016), we extended the traditional framework of vertex models and introduced four distinct ‘stripes’ of cell identities within each parasegment. Each cell in the tissue is assigned an index $\alpha$ and stripe identity, $S_{\alpha}\in\left\{ 1,2,3,4 \right\}$, where a parasegment contains cells of each identity type (see Fig 3E in Tetley et al., 2016 and S9 Fig). Cells preferentially neighbour cells of the same identity and compartmental boundaries are given by boundaries between cells of differing identities. This is modelled by modifying the line tension of shared edges between cells of different identities, representing the increased junctional contractility observed at compartmental boundaries (see below and (Tetley et al., 2016)).

**Governing equations**

We describe the planar epithelial sheet by a set of $N_{c}$ cells sharing $N_{v}$ vertices connected by $N_{e}$ straight edges representing cell cortices. Each of these variables may change over time due to the plastic rearrangements described below. The elastic mechanical properties of the cells under vertex models are usually characterised in terms of a preferred area, $\tilde{A}_{0}$, relative to a bulk stiffness, $\tilde{K}$, and a preferred perimeter, $\tilde{P}_{0}$, relative to a cortical stiffness, $\tilde{\Gamma}$. Scaling all distances on $\sqrt{\tilde{A}_{0}}$, the dimensionless elastic mechanical energy of the tissue, $U$, can be defined as (Tetley et al, 2016)

|  | $U= \sum_{\alpha}^{N_{c}} \left[ \left( A_{\alpha}- 1 \right)^{2}+\frac{\Gamma}{2}{P_{\alpha}}^{2} \right]+\sum_{(ij)}^{N_{e}} f\left( \left\{ S_{\alpha},S_{\beta} \right\}_{ij} \right).$ | $\sum\left( 1 \right)(1)$ |
| --- | --- | --- |

Here the dimensionless parameter $\Gamma=\tilde{\Gamma}/(\tilde{K}\tilde{A}_{0})$ represents the stiffness of the cortex relative to the bulk, while $A_{\alpha}$ and $P_{\alpha}$ denote the dimensionless area and perimeter of cell $\alpha$, respectively. The first sum runs over all cells. The second sum runs over all edges, ($ij)$ (defined by two vertices $i$ and $j$), contributing to line tensions at cell-cell interfaces, as a function of the stripe identities, $\left\{ S_{\alpha},S_{\beta} \right\}_{ij}$, of the cells, $\{\alpha,\beta\}$, sharing the edge, and is given by

|  | $f\left( \left\{ S_{\alpha},S_{\beta} \right\}_{ij} \right)=\left\{ \begin{aligned} \Lambda l_{ij}, &\mathrm{if}S_{\alpha}=S_{\beta} (non\text{-}boundary interfaces) \\ 2\Lambda log{(L}_{\alpha,\beta}), &\mathrm{if}{(S}_{\alpha} mod 4) {=(S}_{\beta} mod 4)+1 (boundary interfaces) \\ 8\Lambda log{(L}_{\alpha,\beta}), &\mathrm{if}{(S}_{\alpha} mod 4) {=(S}_{\beta} mod 4)+2 (mismatched boundary interfaces). \end{aligned} \right.$ | $(2)$ |
| --- | --- | --- |

Here $\Lambda$ is a dimensionless constant, analogous to the line tension term defined in (Farhadifar et al., 2007), while $l_{ij}$ denotes the length of edge ($ij)$ and $L_{\alpha,\beta}$ denotes the largest contiguous length of edges from cell $\alpha$ or $\beta$ connected to the boundary $\left\{ S_{\alpha},S_{\beta} \right\}$ (see Fig 7E in (Tetley et al., 2016). For edges comprised of cells of the same stripe identity ($S_{\alpha}=S_{\beta}$), this line tension expression is a traditional linear function of the edge length, $l_{ij}$. For cells of differing stripe identities ($S_{\alpha}\neq S_{\beta}$), the line tension is super-contractile in two cases: (i) where stripe identities differ by 1 (defined as boundary interfaces), the contractility increases by a factor of 2; (ii) where the identities differ by 2 (defined as mis-matched boundary interfaces), we assume that edges are even more contractile and increase the line tension by a factor of 8. This super-contractility is believed to be a result of anisotropic myosin II localisation downstream of cell-cell interactions (Tetley et al., 2016). The length-dependent contractility at differing stripe identities is also modified to increase exponentially as the edges shorten (this is a requirement under the vertex model to mimic the ratchet-like shortening of cell edges (Rauzi et al., 2010)).

We take a standard approach to compute forces acting on cell vertices directly from the first variation of the mechanical energy (Farhadifar et al., 2007; Nestor-Bergmann et al., 2018a). Noting that $\delta_{i}U=-\boldsymbol{\nabla}_{i}U\cdot\delta\boldsymbol{r}_{i}$, where $\boldsymbol{r}_{i}$ is the position of vertex $i$, we can interpret ${-\boldsymbol{\nabla}}_{i}U$ as the force required to displace vertex $i$ by $\delta\boldsymbol{r}_{i}$. We assume that the motion of vertices is overdamped and model their deterministic evolution by assigning a drag force of the form $\tilde{\eta}\frac{d{\tilde{\boldsymbol{r}}}_{i}(\tilde{t})}{d\tilde{t}}$ (in dimensional units) such that the total dimensionless force, $\boldsymbol{f}_{i}$, acting on vertex $i$ is given by

|  | $\boldsymbol{f}_{i}= -\boldsymbol{\nabla}_{i}U- \frac{d\boldsymbol{r}_{i}\left( t \right)}{dt},$ | $\frac{d\boldsymbol{r}_{i}\left( t \right)}{dt}(3)$ |
| --- | --- | --- |

where time has been rescaled relative to the viscosity, $\tilde{\eta}$ (see (Nestor-Bergmann et al., 2018a) for detailed scalings). In equilibrium $\boldsymbol{f}_{i}=\boldsymbol{0}.$

**Cell intercalation, higher-order vertex formation and resolution**

In addition to solving the equations of motion for cell vertices, we must ensure that cells are always non-intersecting and that they can form and break bonds. In the majority of published vertex models, this is achieved through an elementary operation called a ‘T1 swap’, which corresponds biologically to a cell neighbour exchange (Fig 7A) (Alt et al., 2017; Fletcher et al., 2014). Mathematically, such arrangements represent plastic deformations of the tissue that can decrease the total mechanical energy. We generalise this approach to model the formation and resolution of the experimentally observed apical ‘gaps’ arising in wild-type and *sdk* mutant tissues (Fig 5). Although apical gaps are likely due to a decrease of apical connectivity between cells, this is not formally possible in current vertex models, so we instead describe them in a similar manner to multicellular rosettes (Trichas et al, 2012). We implement this as follows:

*Formation of a four-way vertex:* A four-way vertex is formed whenever two vertices $i$ and $j$ of rank 3 (defined as the number of cells sharing the vertex) are located less than a minimum threshold distance $d_{min}$ apart (taken to be much smaller than a typical cell diameter). In this case, we merge the two vertices into a single vertex located at their midpoint and all cells previously connected to $i$ and $j$ now share a common vertex of rank 4 ().

*Rosette vertex rank increase:* Extending the principle of 4-way vertices, we allow for a vertex of rank $m$ to merge with an existing vertex of rank $n$ to form a hole of rank $n+m-2$. This occurs whenever two vertices $i$ and $j$ (not both having degree 3) are located less than a minimum threshold distance $d_{min}$ apart. In this case, the vertex with higher degree (or, if their degrees are equal, a randomly chosen vertex) remains in position, while the other vertex is merged into it Fig 7C). Vertices with rank greater than 4 are termed rosette vertices.

*Rosette vertex rank decrease:* We allow cells to split off from a vertex or rank greater than 3, reducing its rank by one, as follows. We define a parameter, $p_{5+}$, representing the probability per timestep that a cell will split from a vertex. For each vertex of rank greater than 4 present at each time step, we generate a random number uniformly from $U[0,1]$. If this number exceeds $p_{5+}$, we randomly select one cell connected to the vertex, then create a new vertex along the line joining the vertex and the centroid of this cell, at a distance ${\lambda d}_{min}$, and update cells adjacent to this cell to incorporate this new vertex. We continue this process for each vertex of rank greater than 4 in the tissue at this time step, including any that have already decreased their rank at this time step (this allows for the possibility of several cells breaking off from a vertex in the same time step and ensures that the rate of vertex resolution is independent of time step size; Fig 7C).

We deal with resolution of rank-4 vertices slightly differently, considering these as the ‘completion’ of a T1 swap. For each vertex of rank 4 present at each time step, we generate a random number uniformly from $U[0,1]$. If this number exceeds $p_{4}$, we randomly select one cell containing the vertex, and identify the cell opposite it across the vertex, create a new vertex along the line joining the vertex and the centroid of the randomly selected cell, at a distance ${\lambda d}_{min}$, and move the existing vertex the same distance along the line joining its original position and the centroid of the opposite cell. We then update all cells adjacent to the randomly selected cell to incorporate this new vertex (Fig 7B). Note that $p_{4}\neq p_{5+}$ in general, as we observe experimentally that higher-order gaps have a longer persistence time (Fig 5F). However, we do not distinguish between the persistence time of rosette vertices greater than rank 5 and approximate all of these with $p_{5+}$.

In a departure from previous vertex models of cell rearrangement, we do not force cell rearrangements through T1 swaps on short edges. Instead, we consider all cell rearrangements as the formation of a rank-4 vertex with resolution probability $p_{4}$ at every time step. This allows us to account for the time taken to perform a neighbour exchange, and makes it possible that some cells in the process of exchanging neighbours (rank-4 vertices) merge with other cells to form more stable higher-order vertices before the neighbour exchange is completed. The *sdk* mutants are modelled by increasing the persistence time of rank-4 vertices and posing that higher-order vertices are completely stable ($p_{5+}^{SDK}=0$) over the timeframe of germband extension, based on our experimental observations (Fig 5F).

**Boundary conditions**

In contrast to our previously published model of wild-type germband extension (Tetley et al., 2016), we impose doubly periodic boundary conditions within a rectangular domain of width, $X$, and height, $Y$, aligned with the anterior-posterior ($x$-) and dorso-ventral ($y$-) axes, respectively. Enforcing periodicity reduces the contribution of boundary artefacts, allowing smaller tissues to be simulated. Furthermore periodicity ensures that the tissue does not pinch centrally, leading to the bowing effect seen under the free boundary conditions used in (Tetley et al., 2016).

We replicate the boundary conditions of the germband by imposing that the posterior domain of the tissue can push into a neighbouring tissue of elastic modulus $E$. To do this, at each time step we evaluate the tissue-level stress tensor as (Ishihara and Sugimura, 2012; Nestor-Bergmann et al., 2018a; Nestor-Bergmann et al., 2018b)

|  | $\boldsymbol{\sigma}= \sum_{\alpha=1}^{N_{c}} \sum_{j=1}^{N_{v}^{\alpha}} \boldsymbol{r}_{j}^{\alpha}\otimes\boldsymbol{f}_{j}^{\alpha}$ | $\sum_{j=1}^{N_{v}^{\alpha}} \boldsymbol{r}_{j}^{\alpha}\otimes\boldsymbol{f}_{j}^{\alpha} (4)$ |
| --- | --- | --- |

where $\boldsymbol{r}_{j}^{\alpha}$ is the position of vertex $j$ in cell $\alpha$, $\boldsymbol{f}_{j}^{\alpha}$ is the force exerted by cell $\alpha$ on vertex $j$, $N_{v}^{\alpha}$ is the number of vertices belonging to cell $\alpha$ and $N_{c}$ is the number of cells in the tissue. Assuming that the stress is distributed uniformly around the tissue boundary, we calculate the strain, $\varepsilon^{\mathrm{push}}$, of the posterior boundary (oriented in the anterior-posterior direction) as

|  | $\varepsilon^{\mathrm{push}}=\frac{{-\sigma}_{xx}}{E},$ | $\frac{{-\sigma}_{xx}}{E} (5)$ |
| --- | --- | --- |

where $\sigma_{xx}$ is the anterior-posterior component of the stress tensor and the negative sign arises due to this being the stress exerted by the tissue. The posterior boundary nodes of the rectangular domain are then displaced such that the width of the tissue maps as $X\to X(1+\varepsilon^{\mathrm{push}})$. Note that we scale $\varepsilon^{\mathrm{push}}$ by the numerical timestep, $\Delta t$, such that the results are independent of the chosen timestep. The dorso-ventral boundaries of the box deform in an analogous manner to relieve stress in the dorso-ventral axis, where the neighbouring tissues also have elastic modulus $E$. Thus at every time step, after updating vertex positions according to ($3)$, we calculate the tissue-level stress and displace the boundary nodes (and the size of the periodic box accordingly) along the dorsal, ventral and posterior boundaries to relax the tissue-level stress. However, we fix the position of the anterior boundary of the domain as the germband extends mainly in the posterior direction.

With the above boundary conditions, the tissue will intercalate and deform in the presence of anisotropic dorso-ventrally oriented polarised junctional contractility in a manner equivalent to (Tetley et al., 2016).

**Modelling a posterior pull**

In an extension to previous models, we also modelled the presence of an extrinsic pull in the posterior direction, representing the action of the posterior midgut (Butler et al., 2009; Collinet et al., 2015; Lye et al., 2015). This is done in an analogous manner to the pushing of the germ-band into neighbouring tissues described above. However, in this case, we apply a stress, $\sigma^{\mathrm{pull}}$, from the posterior neighbouring tissue and must calculate the elastic resistance from the germ-band to determine the induced strain. This is done numerically at every timestep. We begin by storing the current tissue-level stress tensor, $\boldsymbol{\sigma}^{0},$ then impose a small strain, $\varepsilon_{xx}$, on the posterior nodes of the germ-band and calculating the new tissue-level stress, $\boldsymbol{\sigma}^{*}$. The anterior-posterior component of the tissue-level stiffness tensor is then given by (Nestor-Bergmann et al., 2018b)

|  | $C_{xx}=\frac{\sigma_{xx}^{*}-\sigma_{xx}^{0}}{\varepsilon_{xx}}.$ | $\frac{\sigma_{xx}^{*}-\sigma_{xx}^{0}}{\varepsilon_{xx}} (6)$ |
| --- | --- | --- |

The true strain induced by the imposed posterior stress may then be calculated as

|  | $\varepsilon^{\mathrm{pull}}=\frac{\sigma^{\mathrm{pull}}}{C_{xx}}.$ | $\frac{\sigma^{\mathrm{pull}}}{C_{xx}}. (7)$ |
| --- | --- | --- |

The tissue is then returned to its original configuration, before $\varepsilon_{xx}$ is applied, and $\varepsilon^{\mathrm{pull}}$ is applied to the posterior nodes, such that the width of the tissue maps as $X\to X(1+\varepsilon^{\mathrm{pull}})$. Note that we scale $\sigma^{\mathrm{pull}}$ by the numerical timestep, $\Delta t$, such that the results are independent of the chosen timestep.

**Initial conditions**

We model the movement, shape change and neighbour exchange of a small tissue that is initially comprised of 20 rows and 14 columns of hexagonal cells. We initialise all cells with the same stripe identity. In this case, it can be shown that a regular $Z$-gon is stress-free when its area, $A$, satisfies (Nestor-Bergmann et al., 2018a)

|  | $A-1+ \frac{\Gamma\mu_{Z}^{2}}{2}+\frac{\Lambda\mu_{Z}}{4\sqrt{A}}=0,$ | $\frac{\Gamma\mu_{Z}^{2}}{2}+\frac{\Lambda\mu_{Z}}{4\sqrt{A}} (8)$ |
| --- | --- | --- |

where $\mu_{Z}=2\sqrt{Z\tan(\pi/Z)}$. Thus we initialise the tissue to be stress free at time $t=0$ by setting the area of the hexagons to satisfy $(8)$. We then bestow stripe identities such that there are approximately 3.5 cells per parasegment (as found experimentally in (Tetley et al., 2016), and numerically solve the dynamical system governing the motion of the vertices until time $T$.

**Computational methodology**

In summary, the configuration of the tissue is updated using the following algorithm. Starting from an initial configuration $\boldsymbol{r}_{i}(0)$, we update the state of the system until time $T$ over discrete time steps $\Delta t$. At each time step we: implement any required vertex rank increases and decreases; compute the forces $\boldsymbol{F}_{i}$ on each vertex from the free energy $U$; solve the equation of motion for each vertex over the time step numerically, using an explicit Euler method; implement an extrinsic pull and update the size of the rectangular domain by relaxing tissue-level stress; and finally update the positions of all vertices simultaneously.

We implement this model in Chaste, an open source C++ library that allows for the simulation of vertex models (Fletcher et al., 2013). The test and source files used in these simulations can be downloaded from <https://github.com/Alexander-Nestor-Bergmann/GBE_vertex_model_sdk>

The values of all parameters used in the simulations are provided in Table 1 at the end of this Supplement.

**Simulations**

Simulation 1 – wild-type tissue (Fig 7E,F)

In the first simulation, we model a wild-type tissue under the influence of contractile cables (via differential line tensions between cell identities) and posterior pulling forces. The maximum external stress, $\sigma_{max}^{\mathrm{pull}}$, at the posterior boundary is set to $7\times{10}^{-5}\Delta t$ (scaled by the timestep). However, rather than applying an instantaneous constant stress, we simulate the gradual increase and decrease in the tissue extension strain rates measured in our experiments (Fig 6B) by gradually increasing the pull to a peak at time $t=T/4$ and then decreasing the magnitude. This is done as a simple linear increase to the peak followed by a linear decrease:

|  | $\sigma^{\mathrm{pull}}(t)=\left\{ \begin{aligned} \sigma_{max}^{\mathrm{pull}}\frac{4t}{T}, &t<T/4 \\ \sigma_{max}^{\mathrm{pull}} \left( 1-\frac{t-T/4}{1.25T} \right), &t\geq T/4 \end{aligned} \right.$ | $(9)$ |
| --- | --- | --- |

Note that the pull is not zero at $t=T$. Both the magnitude and delay of the pull were tuned to match the experimental strain rates. As the tissue contracts and extends, its stiffness tensor may change and lead to different responses to $\sigma^{\mathrm{pull}}$ over time. We use Fig 5F to determine order-of-magnitude estimates for the stability of higher-order vertices (their resolution time) in the tissue. Because of the limitation in resolution of the light confocal microscopy used in Fig 5F, only large gaps can be detected by visual inspection, rather than all delayed rosettes. So we set $p_{4}^{WT}$ and $p_{5+}^{WT}$ a factor of 10 larger than the observed values in Fig 5F, which results in final topologies reminiscent to experiments. The anterior-posterior width, $X$, of the periodic box is recorded at every timestep, from which we can calculate the anterior-posterior strain rate (Fig 7F).

Under this new model of cell rearrangement, this simulation results in successful convergent-extension of the germband. The anterior-posterior strain rates are well matched to the experimentally observed curves (compare Fig 6B to Fig 7F).

Simulation 2 – stable rosettes (Fig 7G,H)

Following the observation that gaps persist for longer in *sdk* mutants (Fig 5F), we explored the effect of decreasing the resolution probabilities in simulations. Our hypothesis is that the presence of apical gaps at the centre of 4 cells or rosettes (5 cells and above) indicates that vertex resolution at the level of adherens junctions is delayed or blocked. All simulations parameters were identical to simulation 1, except the resolution probabilities of rank-4 and rank-5 vertices, which were set to $p_{4}^{sdk}=0.1p_{4}^{WT}$ and $p_{5+}^{sdk}=0.$We set these values based on the changes in persistence times in Fig 5F. Indeed, gaps found at the centre of rosettes (5 cells and above) often do not resolve in the timescale of germband extension (see also S6 Fig).

We find that the tissue successfully performs convergent-extension, though with less cumulative tissue-level anterior-posterior strain than wild-type tissue (compare Fig 7E, F to Fig 7G, H). This is likely due to the imposed delay in intercalation through increasing the persistence time of higher-order vertices. However, the initial peak in the strain rate is not reduced, as was observed in experiments (Fig 6B).

Simulation 3 – stable rosettes and mechanical perturbation (Fig 7I, J)

Given the poor matching of the anterior-posterior strain rate between simulation 2 and the experiments, we next explored the possibility that Sidekick also alters the mechanical properties of the tissue. Because Sidekick is known to mediate homophilic adhesion, one possibility is that the intercellular adhesion is decreased to some extent in *sdk* mutants. It has been shown that, for a mechanically homogeneous tissue, the tissue shear modulus can be reduced by reducing the mechanical parameter $\Gamma$ (Nestor-Bergmann et al., 2018b). This results in a concomitant increase of the tissue bulk modulus, placing constraints on how the tissue responds to both internally generated and externally applied stress. We explore how such a change may affect convergent extension by reducing $\Gamma$ by a factor of 4 and keeping all other parameters equivalent to simulation 3.

We find that this mechanical perturbation is sufficient to offset the peak strain rate, in a similar manner to experimental observations (compare Fig 6B to Fig 7J). Interestingly, cell geometries and topologies are severely perturbed, with more distorted polygons (as the cells become less resistant to shear) and far more rosettes, which is also observed in experiments (compare Fig 4A, B to Fig 7E, G, J).

We note that, if there is mechanical perturbation in *sidekick* mutants, the stiffness of neighbouring tissues and the posterior pull may also be altered. However, for simplicity we keep the number of tuneable parameters small and choose not to explore these effects. It is possible that similar results could be achieved by altering $E$ and $\sigma_{max}^{\mathrm{pull}}$.

**Table 1.** The parameters and their values used in the vertex model simulations.

| **Parameter** | **Description** | **Value** | **Reference(s)** | **Simulations used** |
| --- | --- | --- | --- | --- |
| $T$ | Dimensionless simulation end time | 400 | - | 1,2 & 3 |
| $\Delta t$ | Dimensionless timestep | 0.01 | - | 1,2 & 3 |
| $d_{min}$ | Dimensionless T1 swap threshold | 0.01 | (Kursawe et al., 2017) | 1,2 & 3 |
| $\lambda$ | T1 swap distance multiplier | 1.5 | (Kursawe et al., 2017) | 1,2 & 3 |
| $\Gamma$ | Dimensionless contractility coefficient | 0.04 | (Farhadifar et al., 2007) | 1 & 2 |
| $\Gamma^{*}$ | Reduced dimensionless contractility coefficient | $\Gamma/4$ | - | 3 |
| $\Lambda$ | Dimensionless internal line-tension coefficient | 0.05 | (Tetley et al., 2016) | 1,2 & 3 |
| $p_{4}^{WT}$ | Rank 4 hole resolution probability per timestep in wild-type tissue. | $10\Delta t\frac{30}{T}$ | Fig 5F | 1 |
| $p_{5+}^{WT}$ | Rank 5, or more, hole resolution probability per timestep in wild-type tissue. | $\Delta t\frac{30}{T}$ | Fig 5F | 1 |
| $p_{4}^{SDK}$ | Rank 4 hole resolution probability per timestep in *sdk* tissue. | $0.1p_{4}^{WT}$ | Fig 5F | 2 & 3 |
| $p_{5+}^{SDK}$ | Rank 5, or more, hole resolution probability per timestep in *sdk* tissue. | 0 | Fig 5F | 2 & 3 |
| $E$ | Dimensionless elastic modulus of tissues neighbouring germband | 75 | - | 1,2 & 3 |
| $\sigma_{max}^{\mathrm{pull}}$ | Maximum dimensionless stress applied to posterior boundary due to midgut | $7\times{10}^{-5}\Delta t$ | - | 1,2 & 3 |

**References:**

**Alt, S., Ganguly, P. and Salbreux, G.** (2017). Vertex models: from cell mechanics to tissue morphogenesis. *Phil. Trans. R. Soc. BPhil. Trans. R. Soc. B* **372**.

**Butler, L. C., Blanchard, G. B., Kabla, A. J., Lawrence, N. J., Welchman, D. P., Mahadevan, L., Adams, R. J. and Sanson, B.** (2009). Cell shape changes indicate a role for extrinsic tensile forces in Drosophila germ-band extension. *Nat Cell Biol* **11**, 859-864.

**Collinet, C., Rauzi, M., Lenne, P. F. and Lecuit, T.** (2015). Local and tissue-scale forces drive oriented junction growth during tissue extension. *Nat Cell Biol* **17**, 1247-1258.

**Farhadifar, R., Roper, J. C., Aigouy, B., Eaton, S. and Julicher, F.** (2007). The influence of cell mechanics, cell-cell interactions, and proliferation on epithelial packing. *Curr Biol* **17**, 2095-2104.

**Fletcher, A. G., Osborne, J. M., Maini, P. K. and Gavaghan, D. J.** (2013). Implementing vertex dynamics models of cell populations in biology within a consistent computational framework. *Prog Biophys Mol Biol* **113**, 299-326.

**Fletcher, A. G., Osterfield, M., Baker, R. E. and Shvartsman, S. Y.** (2014). Vertex models of epithelial morphogenesis. *Biophysical journal* **106**, 2291-2304.

**Ishihara, S. and Sugimura, K.** (2012). Bayesian inference of force dynamics during morphogenesis. *J Theor Biol* **313**, 201-211.

**Kursawe, J., Baker, R. and Fletcher, A.** (2017). Impact of implementation choices on quantitative predictions of cell-based computational models. *Journal of Computational Physics* **345**, 752-767.

**Lye, C. M., Blanchard, G. B., Naylor, H. W., Muresan, L., Huisken, J., Adams, R. J. and Sanson, B.** (2015). Mechanical Coupling between Endoderm Invagination and Axis Extension in Drosophila. *PLoS Biol* **13**, e1002292.

**Nestor-Bergmann, A., Goddard, G., Woolner, S. and Jensen, O. E.** (2018a). Relating cell shape and mechanical stress in a spatially disordered epithelium using a vertex-based model. *Mathematical Medicine and Biology: A Journal of the IMA* **35**, i1–i27.

**Nestor-Bergmann, A., Johns, E., Woolner, S. and Jensen, O. E.** (2018b). Mechanical characterization of disordered and anisotropic cellular monolayers. *Phys. Rev.* **97**, 052409.

**Rauzi, M., Lenne, P. F. and Lecuit, T.** (2010). Planar polarized actomyosin contractile flows control epithelial junction remodelling. *Nature* **468**, 1110-1114.

**Tetley, R. J., Blanchard, G. B., Fletcher, A. G., Adams, R. J. and Sanson, B.** (2016). Unipolar distributions of junctional Myosin II identify cell stripe boundaries that drive cell intercalation throughout Drosophila axis extension. *Elife* **5**, e12094.
