# Supplementary material for: The tricellular vertex-specific adhesion molecule Sidekick facilitates polarised cell intercalation during Drosophila axis extension
Source: PLoS Biol. 2019 Dec 5;17(12):e3000522. doi: 10.1371/journal.pbio.3000522 (PMC6894751; doi:10.1371/journal.pbio.3000522)
Supplement: S1 Table — Sdk, Sidekick; YFP, yellow fluorescent protein. (DOCX) [file pbio.3000522.s007.docx]

| **Stage** | **Epithelial Tissue** | **Morphology** | **Septate junction?** | **Sdk-YFP expressed?** | **Sdk-YFP localisation** |
| --- | --- | --- | --- | --- | --- |
| **Embryo** | Ectoderm early  (< stage 12) | Columnar monolayer | Immature | Yes | Apical vertices  (also appears bicellular during GBE) |
|  | Ectoderm late  (> stage 12) | Columnar monolayer | Mature | Yes | Apical vertices |
|  | Amnioserosa  (stages 11-13) | Squamous monolayer | Immature | Yes | Apical vertices |
|  | Hindgut (stage 13) | Cuboidal monolayer | Mature | Yes | Apical vertices |
|  | Salivary glands (stage 13) | Columnar monolayer | Mature | Yes | Apical vertices |
| **Third Instar Larvae** | Eye-antennal imaginal disc | Columnar monolayer | Mature | Yes | Apical vertices |
|  | Peripodial membrane, imaginal discs | Squamous monolayer | Mature | Yes | Apical vertices |
|  | Limb imaginal disc proper | Pseudostratified monolayer | Mature | Yes | Apical vertices |
|  | Salivary glands | Cuboidal monolayer | Mature | Yes | All lateral (strong) and basal membranes (weak) |
|  | Trachea | Cuboidal monolayer | Mature | Yes | Apical vertices |
|  | Follicular epithelium (before stage 8) | Cuboidal monolayer | Immature | Yes | Apical vertices |
| **Adult** | Midgut | Cuboidal monolayer | Mature | No | n/a |
