# Supplementary figures and images for: The tricellular vertex-specific adhesion molecule Sidekick facilitates polarised cell intercalation during Drosophila axis extension
Source: PLoS Biol. 2019 Dec 5;17(12):e3000522. doi: 10.1371/journal.pbio.3000522 (PMC6894751; doi:10.1371/journal.pbio.3000522)

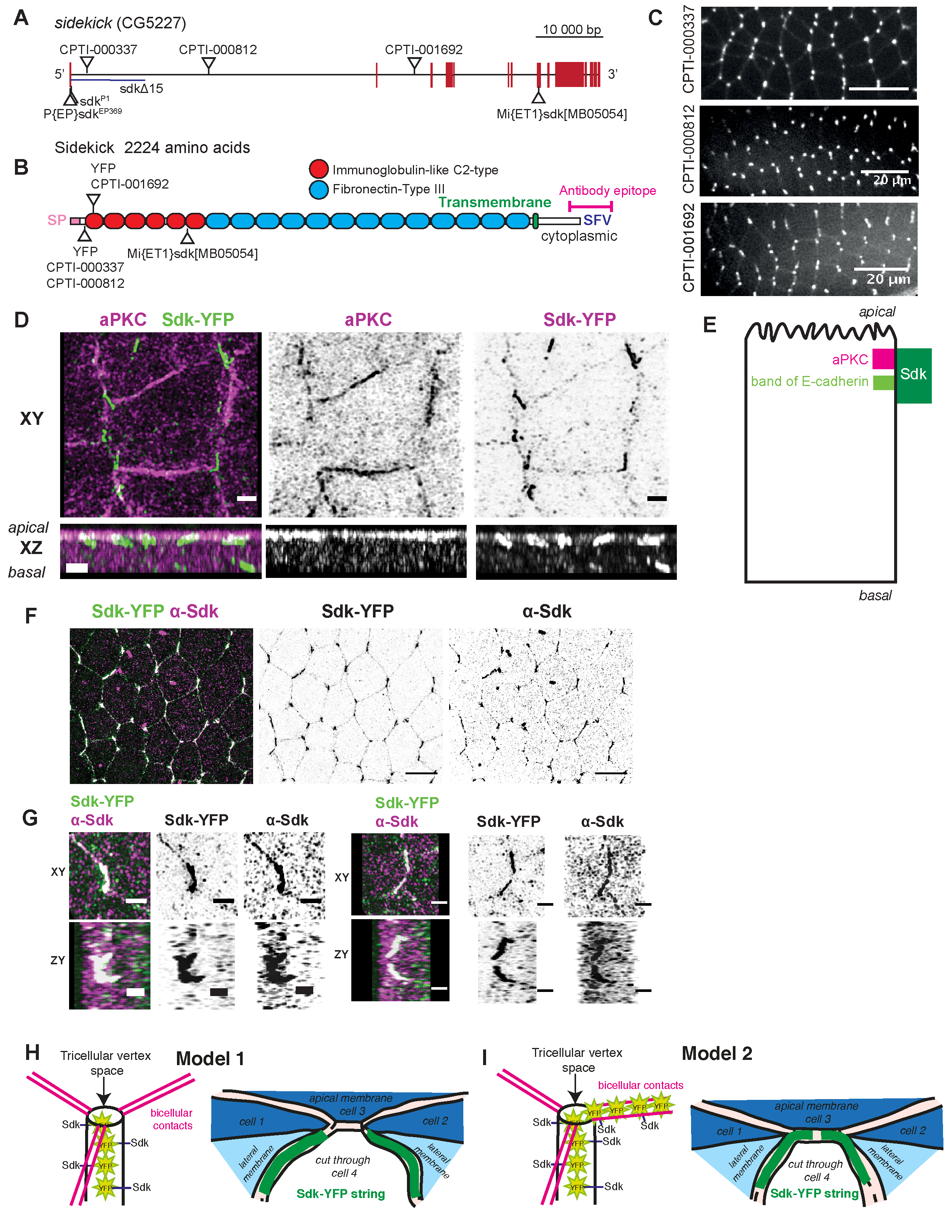

Supplement: S1 Fig — (A and B) Schematics showing the genomic structure of the sdk gene (A) and the domains of the corresponding protein (B). Transposon insertions, alleles, and C-term location of the antibody epitope are indicated. C) All three YFP protein traps from the CPTI collection localise at vertices in the embryonic ectoderm, shown here in images of the ventral embryonic ectoderm in live embryos, taken by Claire Lye and Huw Naylor during our CPTI screen [19]. Scale bar = 20 μm. (D) Super-resolution SIM imaging of fixed embryos immunostained with Sdk-YFP and aPKC. Maximum projection (XY) and z-reconstruction (XZ). Scale bars = 1 μm. (E) Cartoon summarising the apicobasal localisation of Sdk in Drosophila epithelia based on SIM imaging in D. (F, G) Super-resolution SIM imaging of fixed embryos immunostained with Sdk-YFP and an antibody recognising a C-term epitope in Sdk [26]. (F) Maximum projection, apical view. Scale bars = 5 μm. (G) Close-ups of individual strings to show the colocalisation between Sdk-YFP and the Sdk antibody signal. Alignment between channels for super-resolution imaging was performed with the help of fluorescent beads. Scale bars = 1 μm. (H) In model 1, Sdk-YFP remains at tricellular contacts, and protrusions containing Sdk-YFP follow the shortening contact, explaining its apparent localisation at shortening junctions. (I) Alternatively, in model 2, Sdk-YFP molecules do not remain tricellular and invade the bicellular contact at shortening junctions. aPKC, Atypical protein kinase C; CPTI, Cambridge Protein Trap Insertion; Sdk, Sidekick; SIM, Structured Illumination Microscopy; tAJ, tricellular adherens junction; YFP, yellow fluorescent protein. (TIF) [file pbio.3000522.s008.tif]

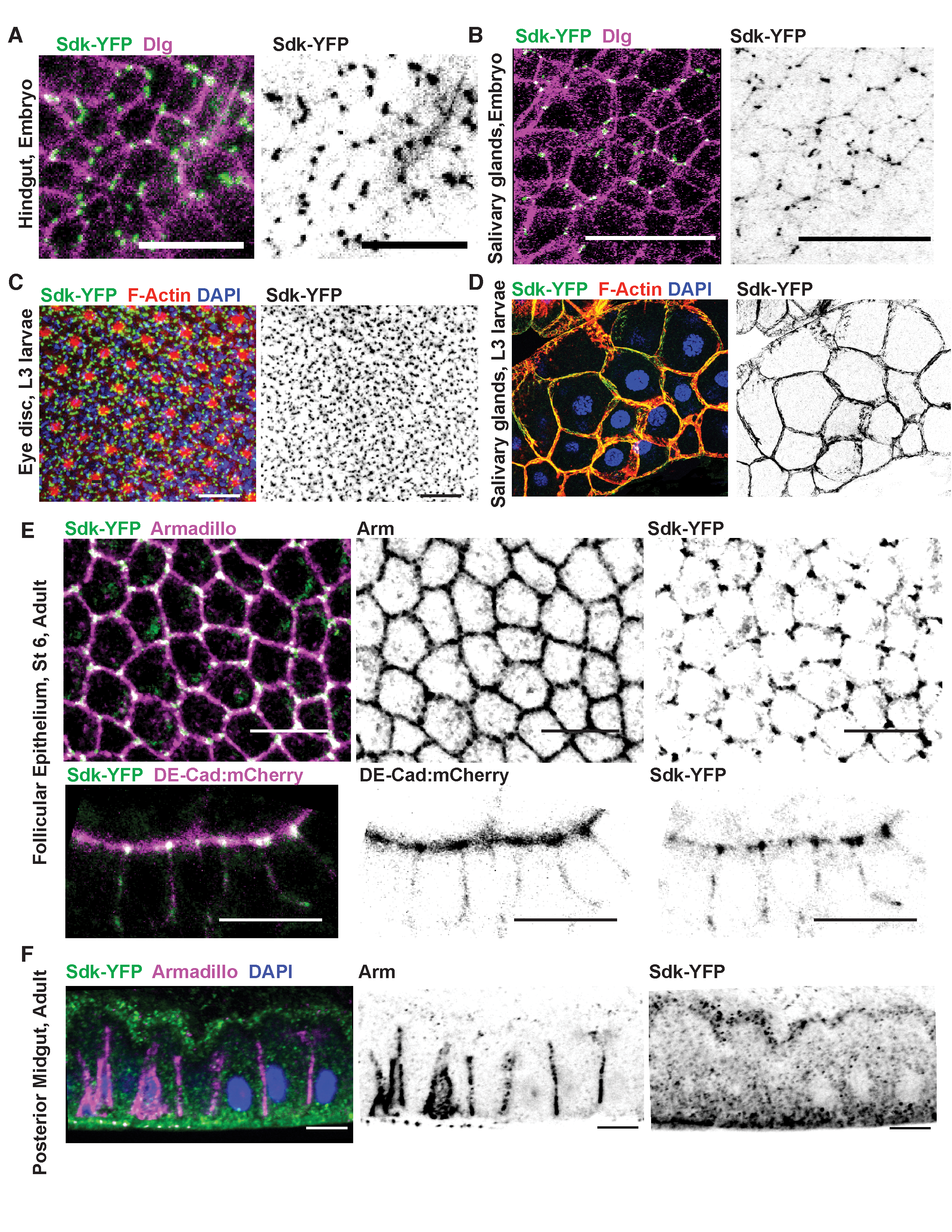

Supplement: S2 Fig — Images show stainings or live imaging of Sdk-YFP in diverse epithelia from different developmental stages. (A) Hindgut, stage 13 embryo, fixed and immunostained tissue, maximum intensity projection. (B) Salivary glands, stage 13 embryo, fixed and immunostained tissue, maximum intensity projection. (C) Eye imaginal disc posterior to the morphogenetic furrow. Dissected from third instar wandering larvae. Fixed and immunostained tissue, maximum intensity projection. (D) Salivary gland. Dissected from third instar wandering larvae. In this tissue, Sdk-YFP localises to all lateral and basal cell–cell junctions. Fixed and immunostained tissue, maximum intensity projection. (E) Follicular epithelium from stage 6 egg chamber from ovaries of adult female flies. Sdk-YFP localises to apical vertices at mitotic stages. Live imaging. Top: apical view, maximum intensity projection. Bottom: lateral view, single z-slice. (F) Posterior midgut of 3-day–old adult female flies. Fixed and immunostained tissue, lateral view, single z-slice. All scale bars = 20 μm. Sdk, Sidekick; YFP, yellow fluorescent protein. (TIF) [file pbio.3000522.s009.tif]

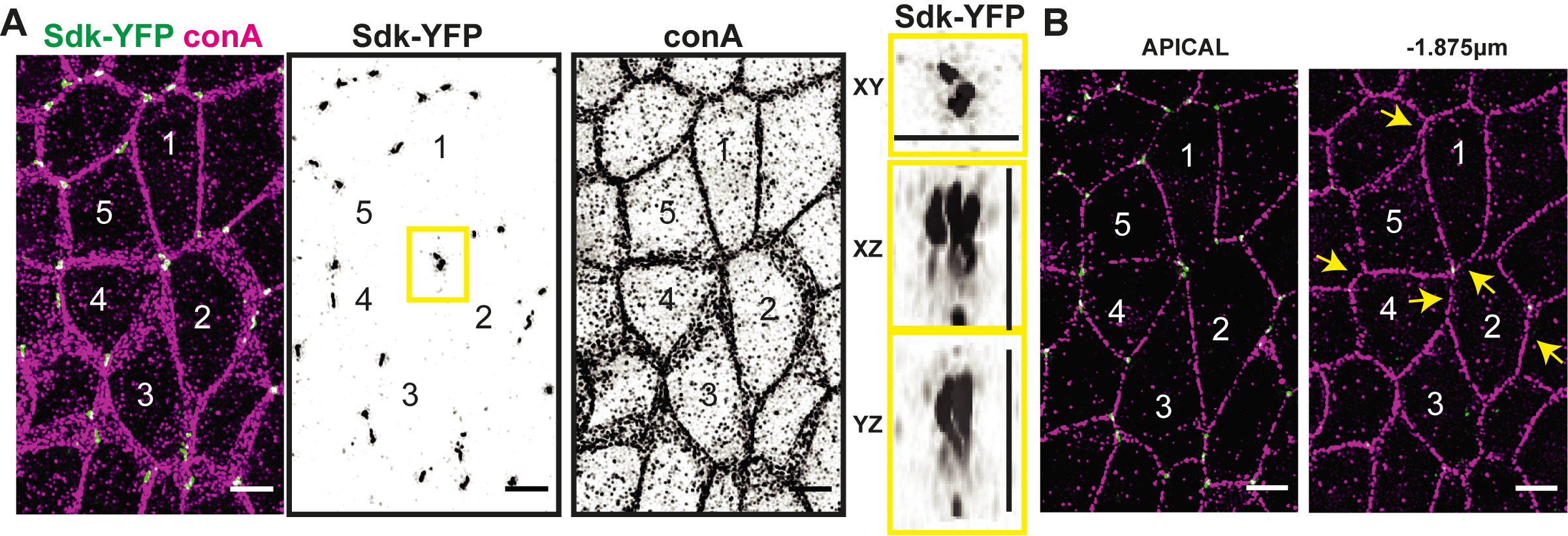

Supplement: S3 Fig — (A) Sdk-YFP string localisation at a rosette centre involving five cells, imaged by super-resolution SIM. The image is from a stage 8 embryo fixed and stained for GFP and the leptin Concanavalin A, a membrane binding protein. Maximum projection over 15 slices = 1.875 μm. Close-ups of the rosette centre with different projections are shown in yellow boxes to demonstrate that three distinct strings can be resolved in the apical-most projections. (B) Single z-slices of the stack shown in A at different apicobasal depths. Sdk-YFP strings represent the apical-most organisation of junctions. Yellow arrows point to junctions that have a different configuration in the z-slice 1.875 μm more basal. All scale bars = 2 μm (including in close-ups). GFP, green fluorescent protein; Sdk, Sidekick; SIM, Structured Illumination Microscopy; YFP, yellow fluorescent protein. (TIF) [file pbio.3000522.s010.tif]

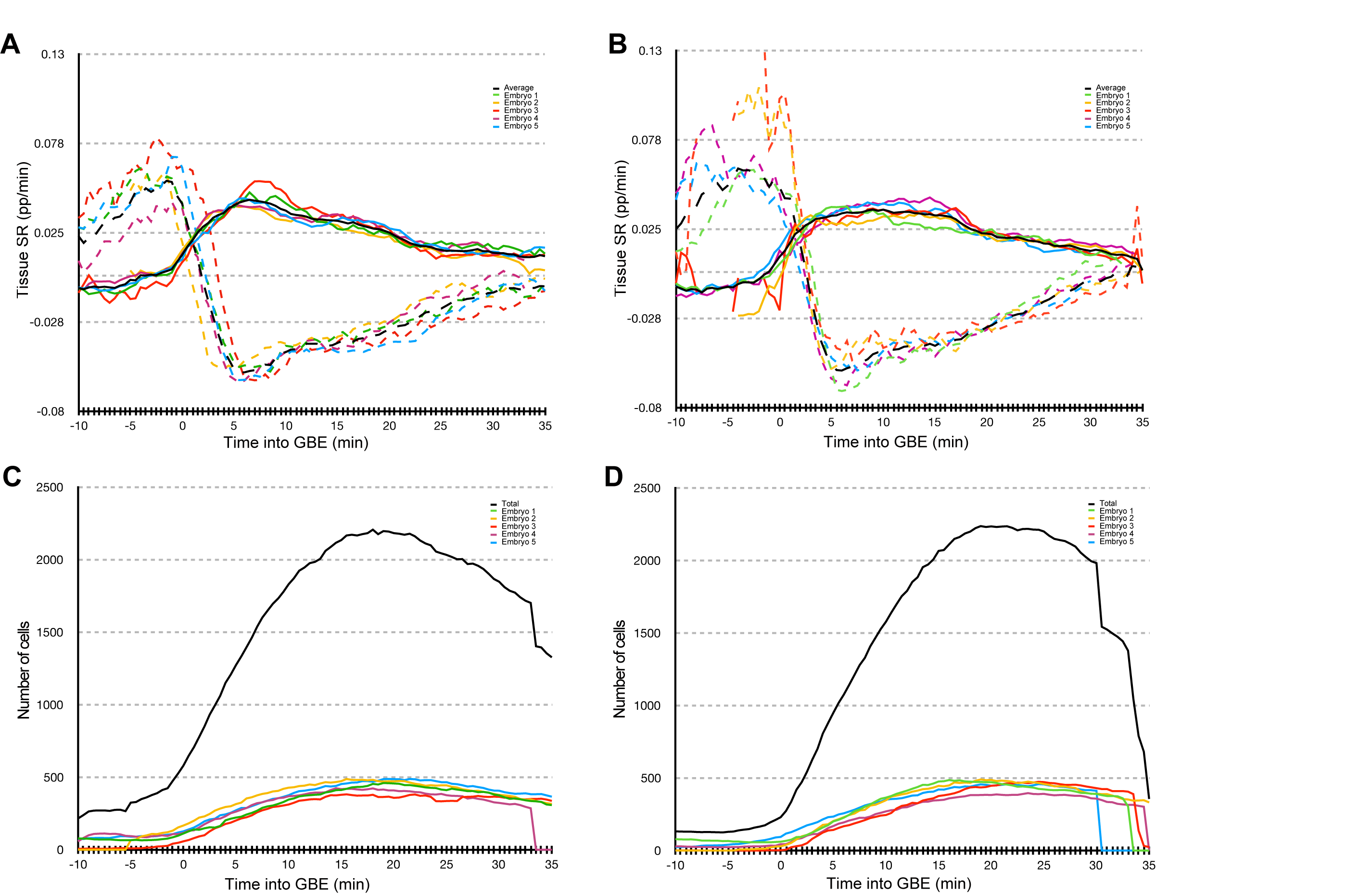

Supplement: S4 Fig — (A–B) Summary of tissue deformation (strain) rates for five wild-type (A) and five sdk (B) embryos in the course of GBE. Tissue strain rates are plotted for both tissue extension along AP (full curves) and convergence along DV (dashed curves). All movies are synchronised to a time point corresponding to the extension strain rate first exceeding 0.01 (proportion per minute), which defines time 0 of GBE. In analyses throughout the paper, we summarise data for the first 30 minutes of GBE. Note that the positive deformation in DV (dotted curves) around the start of extension is due to the ectoderm tissue being pulled ventrally by mesoderm invagination. Averaged data between all five movies are shown as black curves for each genotype. (C,D) Numbers of cells tracked then selected for analysis for each wild-type and sdk movie (total cell number for each genotype in shown in black). The number of successfully tracked cells is low at the onset of GBE because fewer ventral ectodermal cells are in view because of mesoderm invagination. Data for graphs can be found at https://doi.org/10.17863/CAM.44798. AP, anteroposterior; DV, dorsoventral; GBE, germband extension; Sdk, Sidekick. (TIF) [file pbio.3000522.s011.tif]

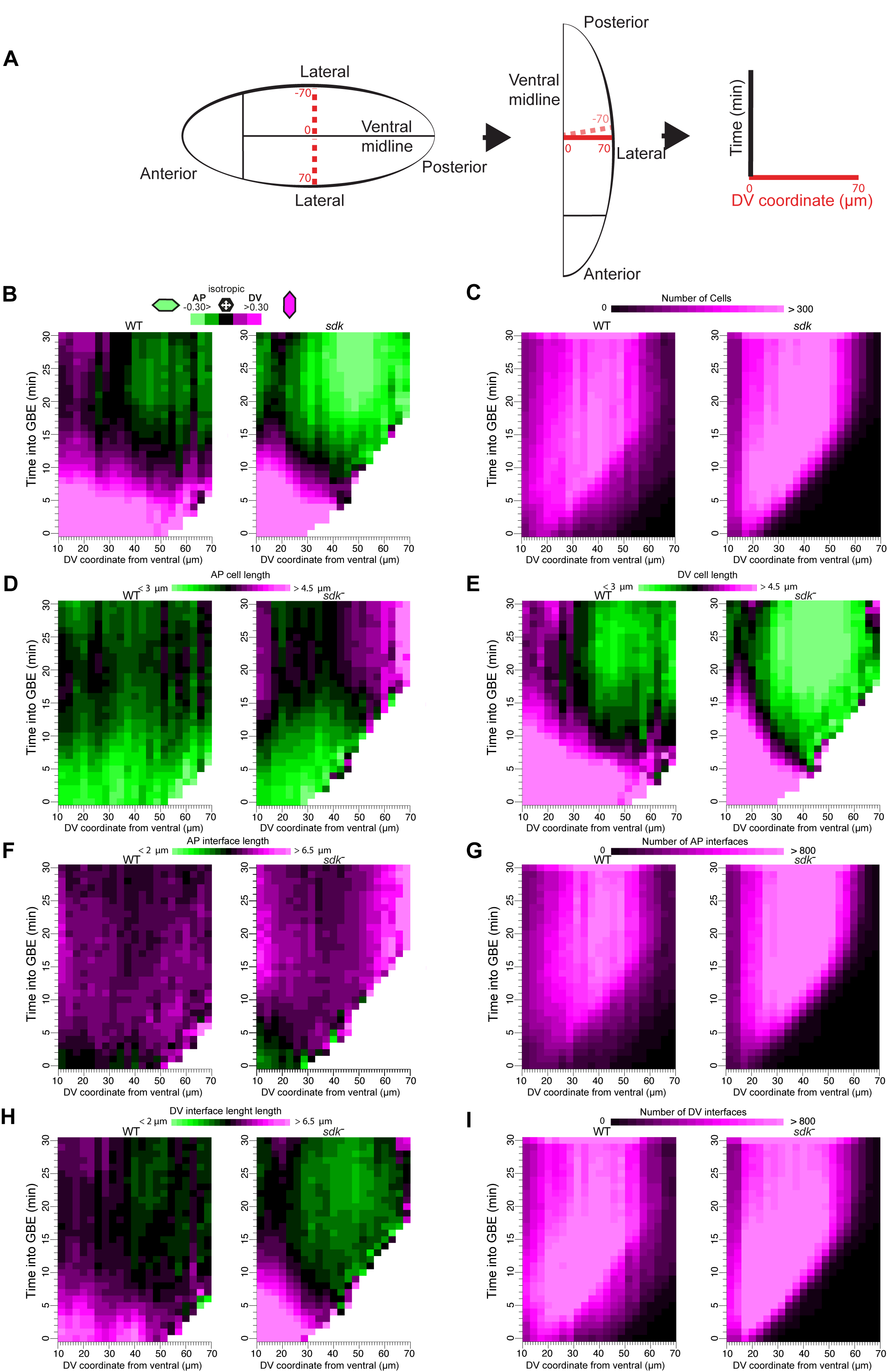

Supplement: S5 Fig — (A) Coordinate system used for spatiotemporal plots shown in B–I. Spatial data are collapsed along AP and given as a function of location along the DV axis. Locations are indicated in μm from the ventral midline (0 at the midline, up to 70 μm laterally). Because of bilateral symmetry, we can mirror the data from the two halves of the embryo along the midline. This simplifies the DV coordinates, and we use x-axes showing locations from 10 to 70 μm. The y-axis gives the time from GBE onset. (B) Evolution of axial shape elongation (see also Fig 4C and 4D) for WT and sdk for the first 30 mins of GBE (y-axis) and as a function of cell position along DV (x-axis). (C) Number of analysed cells per bin for the same spatiotemporal parameters, for graphs B, D, E. (D,E) Spatiotemporal evolution of AP or DV cell length (see also Fig 4E and 4F). (F–I) Spatiotemporal evolution of the lengths of AP or DV cell interfaces (see also Fig 4G and 4H). G and I give the number of AP or DV cell interfaces analysed for each spatiotemporal bin. Note that the raw data shown in all above panels are summarised in Fig 4D–4H. AP, anteroposterior; DV, dorsoventral; GBE, germband extension; Sdk, Sidekick; WT, wild type. (TIF) [file pbio.3000522.s012.tif]

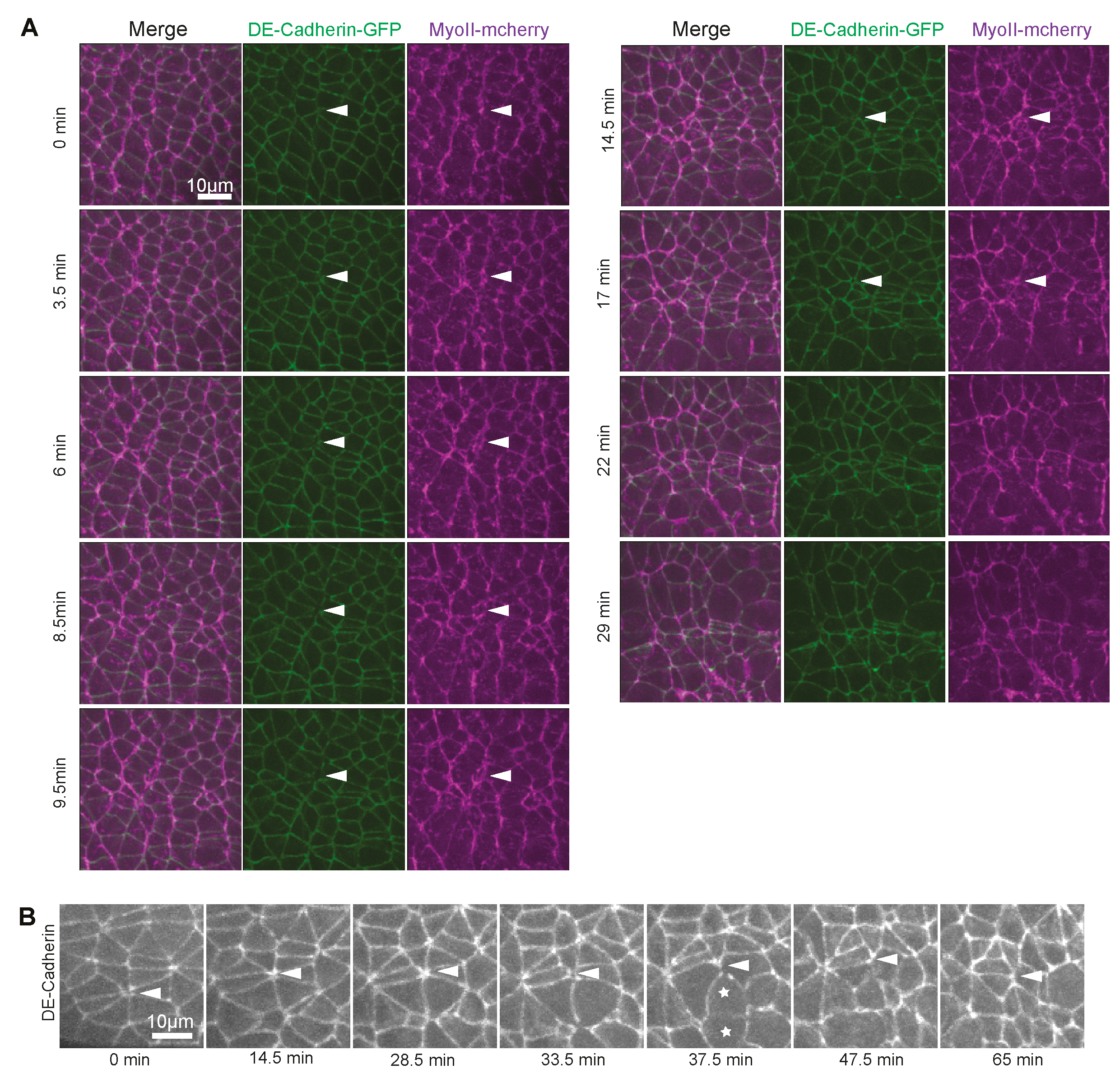

Supplement: S6 Fig — (A) Representative example of the formation and resolution of an apical gap in an sdk mutant embryo labelled with DE-Cadherin and MyoII-Cherry. A projection of 3 μm (± 1 μm from AJ) is shown for each time point. (B) Representative example of a persistent apical gap in an sdk mutant embryo that is finally resolved when cells nearby the gap start dividing (stars marks dividing cells). AJ, adherens junction; MyoII, Myosin II; Sdk, Sidekick. (TIF) [file pbio.3000522.s013.tif]

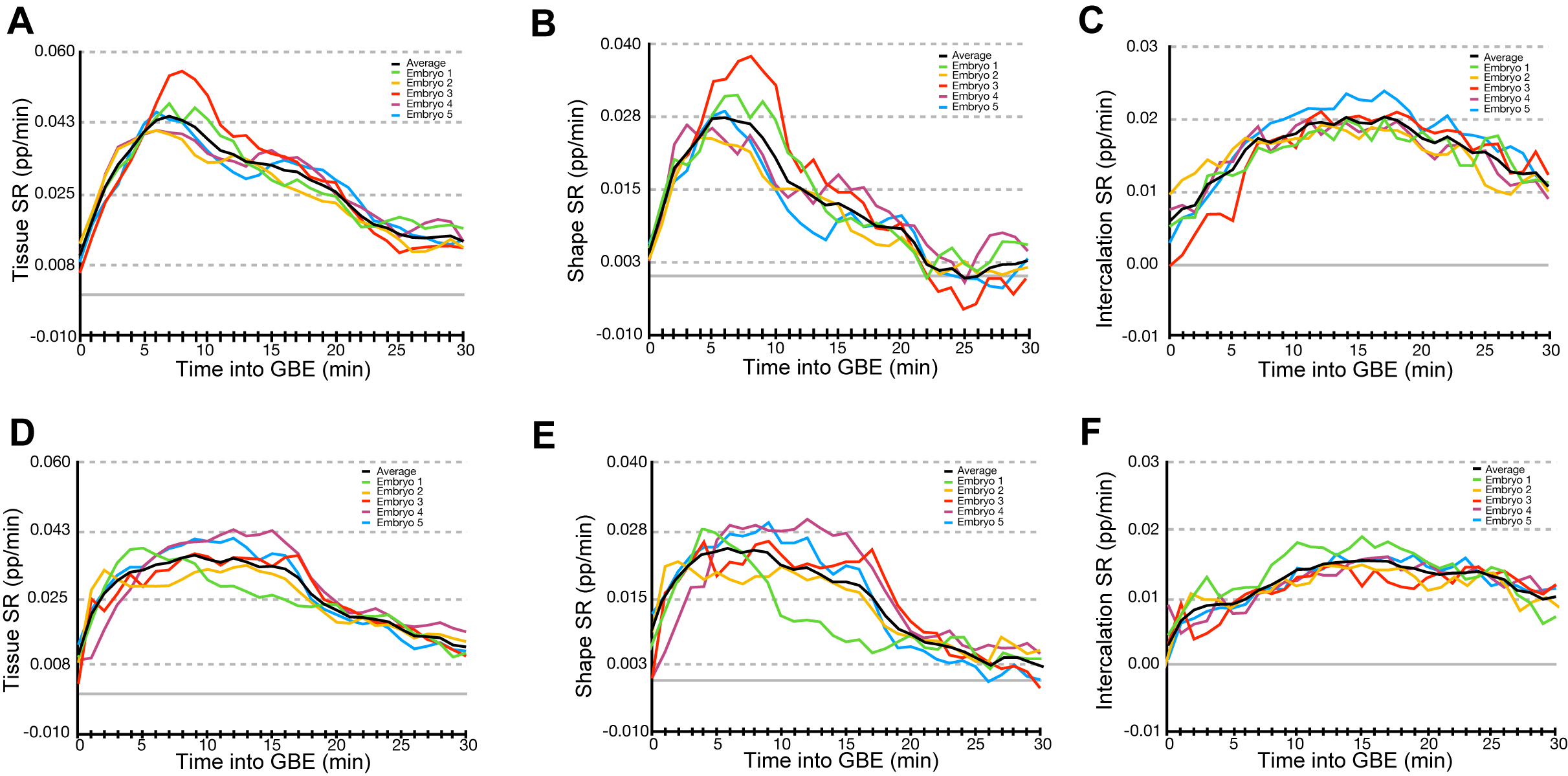

Supplement: S7 Fig — (A–C) Strain rates for each of five wild-type movies. (D–F) Strain rates for each of five sdk movies. (A,D) Total tissue strain rates. (B,E) Cell shape strain rates. (C,F) Cell intercalation strain rates. All movies are labelled with ubi-E-Cad-GFP (see Materials and Methods). Strain rates are along AP, the direction of tissue extension, and are given in pp per minute for the first 30 minutes of GBE. The average for each genotype is shown as a black curve. Data for graphs can be found at https://doi.org/10.17863/CAM.44798. AP, anteroposterior; E-Cad, E-Cadherin; GBE, germband extension; GFP, green fluorescent protein; pp, proportion; Sdk, Sidekick; ubi, Ubiquitin. (TIF) [file pbio.3000522.s014.tif]

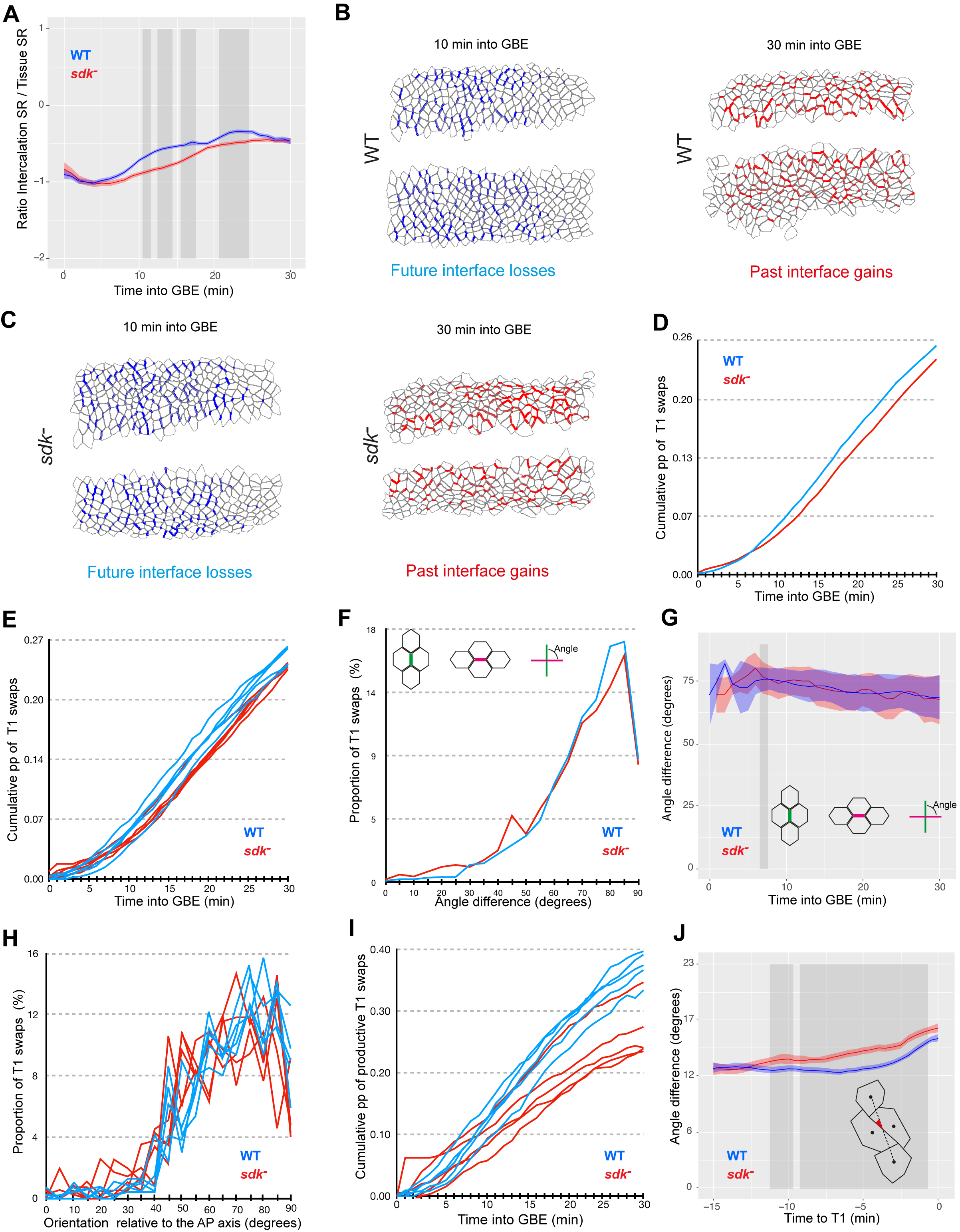

Supplement: S8 Fig — For all graphs, data shown are from the analysis of five wild-type and five sdk mutant embryos (see Materials and Methods). (A) Ratio of cell intercalation/tissue strain rates in AP in wild-type and sdk mutant embryos (see also Fig 6B–6D). (B–C) Detection of T1 swaps in tracked movies for a wild-type (B) and an sdk mutant embryo (C). Movie frames at 10 and 30 minutes into GBE show the cell interfaces that will be lost (blue) and gained (red), respectively, for the detected T1 swaps. (D–E) Cumulative curve of T1 swaps in any direction for the first 30 mins of GBE, expressed as a pp of all cell interfaces tracked at each time point. Average curves for wild-type and sdk embryos (D) and individual curves for each movie (E). (F) Angle between lost and gained cell interfaces during a T1 swap for the first 30 minutes of GBE. The orientation of cell interfaces is measured 5 minutes before and after a swap, respectively. (G) Same quantification as (F) but over the first 30 mins of GBE (x-axis). (H,I) Individual curves for each movie for the quantifications shown in Fig 6F and 6G, respectively. (J) Angle between the shortening cell interfaces in a T1 swap and the line between centroids of the future cell neighbours, as a function of time before swap, in wild-type and sdk mutant embryos. Data for graphs can be found at https://doi.org/10.17863/CAM.44798. AP, anteroposterior; GBE, germband extension; Sdk, Sidekick (TIF) [file pbio.3000522.s015.tif]

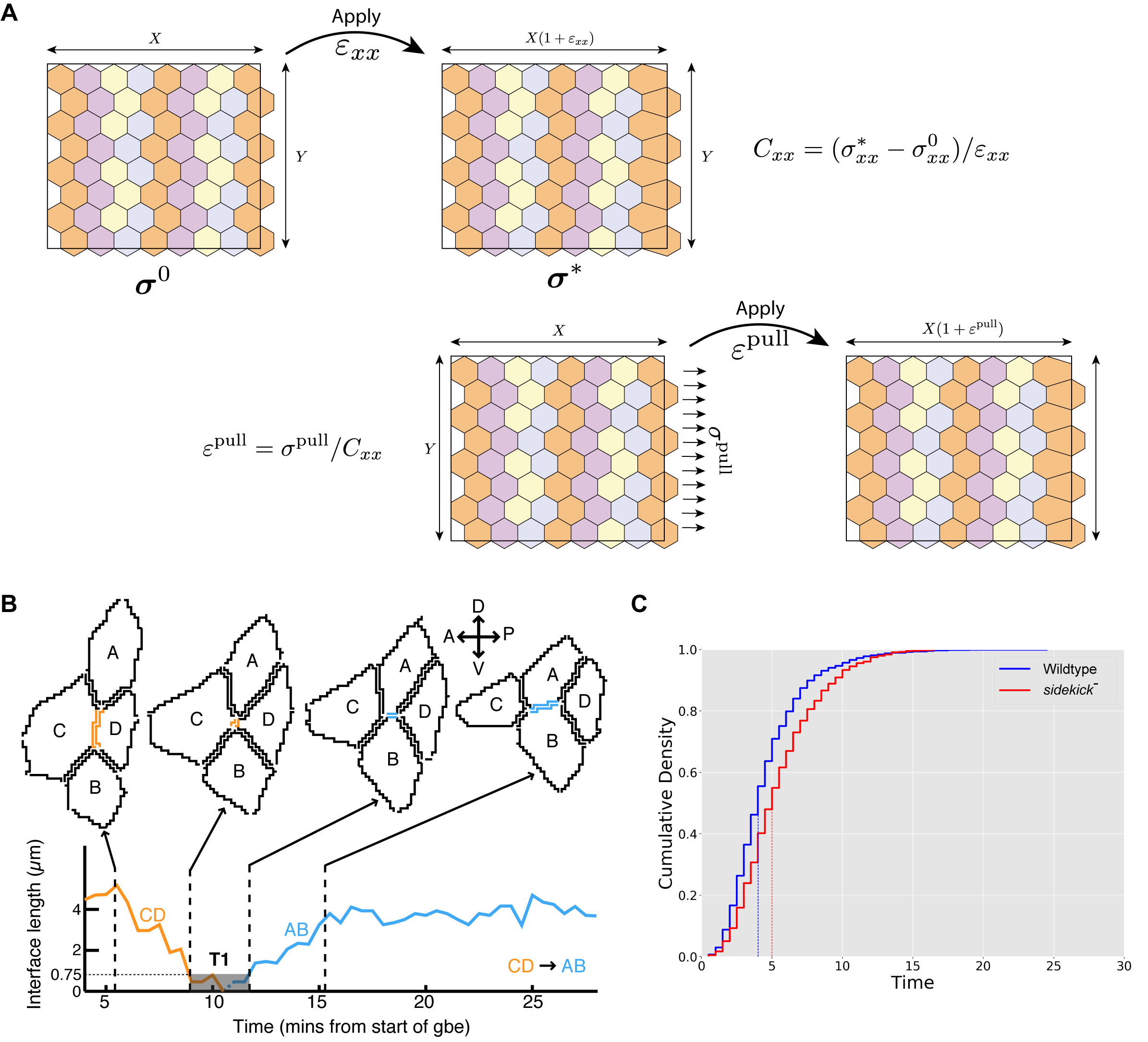

Supplement: S9 Fig — (A) Visualisation of how the posterior strain is calculated in our vertex models given the applied stress, σposterior. A small deformation, Ex, mapping the x-coordinates of vertices as x→x+Exx, is applied to the posterior nodes of the tissue in its current configuration. The AP component of the tissue stiffness tensor, Cxx, can be calculated as the AP component of the change in tissue-level stress over Ex. The tissue is then reverted back to its original configuration, and the true posterior strain is calculated as Eposterior = σposterior/Cxx, which is applied by mapping x-coordinates of vertices as x→x+Eposteriorx. (B) Example of a captured T1 cell rearrangement event in which the junction between cells C and D shortens and is then replaced by a new junction elongating between cells A and B. We define the resolution phase (grey shading) by the time interval when the shortening and subsequently elongating junctions have a length below 0.75 microns. (C) Cumulative histogram of time spent in the resolution phase for the wild type (blue; n = 1,445) and sdk mutant (red; n = 990) tissues during 0–30 min of GBE. Exchanges that do not resolve by 30 min of GBE (for example, stuck rosettes and late rearrangements) are excluded. Wild-type cells have a median resolution time of 4 min, whereas sdk mutants have a median of 5 min. Kolmogorov–Smirnov test finds significant difference between the distributions (p < 1.45 × 10^−13^). Data for graph in C can be found at https://doi.org/10.17863/CAM.44798. AP, anteroposterior; GBE, germband extension; Sdk, Sidekick. (TIF) [file pbio.3000522.s016.tif]
