# Supplementary material for: The tricellular vertex-specific adhesion molecule Sidekick facilitates polarised cell intercalation during Drosophila axis extension
Source: PLoS Biol. 2019 Dec 5;17(12):e3000522. doi: 10.1371/journal.pbio.3000522 (PMC6894751; doi:10.1371/journal.pbio.3000522)
Supplement: Attachment — Submitted filename: Response to reviewers_edited.docx [file pbio.3000522.s018.docx]

We thank the reviewers for their positive and constructive comments. In response, we have made the following changes in the Figures and supplementary data:

Main Figures:
- Figure 2: Edited panels C-E
- New Figure 3 with panels A, B from Fig S3
- Figure 4: was previously Figure 3
- Figure 5 (previously Figure 4): New panels in C
- Figure 6  (previously Figure 5): Removed panels H, I, J (Voronoi analysis)

- Figure 7 (previously Figure 6): Edited panels D-I

- Previous Figure 7: removed- moved models to Fig. S2

Supplementary Figures:
- Figure S1: H is removed and replaced by old Figure 7 panels, now H and I.
- Figure S3: Panels A and B have been moved to a new Figure 3

- Figure S9: New panels B and C

Supplementary movies:

- S1: example of tracked WT movie

- S2: example of tracked *sdk* mutant movie

- S3: example of WT simulation

- S4: example of *sdk* mutant simulation 1

- S5: example of *sdk* mutant simulation 2

We have also added the following references:

**- Choi, W., Acharya, B. R., Peyret, G., Fardin, M. A., Mege, R. M., Ladoux, B., Yap, A. S., Fanning, A. S. and Peifer, M.** (2016). Remodeling the zonula adherens in response to tension and the role of afadin in this response. *J Cell Biol* **213**, 243-260.

**- Sun, Z., Amourda, C., Shagirov, M., Hara, Y., Saunders, T. E. and Toyama, Y.** (2017). Basolateral protrusion and apical contraction cooperatively drive Drosophila germ-band extension. *Nat Cell Biol* **19**, 375-383.

**- Letizia, A., He, D., Astigarraga, S., Colombelli, J., Hatini, V., Llimargas, M. and Treisman, J. E.** (2019). Sidekick Is a Key Component of Tricellular Adherens Junctions that Acts to Resolve Cell Rearrangements. *Dev Cell* **50**, 313-326 e315.

**- Uechi, H. and Kuranaga, E.** (2019). The Tricellular Junction Protein Sidekick Regulates Vertex Dynamics to Promote Bicellular Junction Extension. *Dev Cell* **50**, 327-338 e325.

Below is a point-by-point response to the comments:

*Reviewer #1: 

Specialized junctional structures at epithelial cell vertices were first described in the 1970s, and more recently a number of their protein components were identified in vertebrates and in Drosophila. However, while several recent studies have focused on tricellular occluding (tight) junctions, which seal the paracellular space at cell vertices in epithelia, essentially no information has been available thus far on the structure and molecular composition of cell vertices at the level of adherens junctions, located adjacent to the occluding junctions. This is an important issue because tricellular junctions (TCJs) are increasingly recognized to play fundamental roles in barrier function, epithelial morphogenesis and tissue homeostasis in development and disease. TCJs integrate mechanical forces in epithelia and act as cell shape sensors that can direct mitotic spindle orientation in dividing epithelial cells. A key prerequisite to understand the dynamics and functions of
TCJs in these processes will be to dissect their components, both at the level of the force-transmitting adherens junctions and the occluding tight junctions at cell vertices. 

In this manuscript Finegan et al. describe the subcellular distribution and function of Sidekick (Sdk), a large adhesion molecule with Immunoglobulin and Fibronectin domain repeats that specifically localizes at cell vertices at the level of adherens junctions in various epithelial tissues of Drosophila. The authors use super-resolution imaging to show that Sdk forms string-like structures that appear to extend from the vertex into the adjacent bicellular spaces. Consistent with its predicted role as a homophilic adhesion molecule, Sdk protein is required in pairs of cells for accumulation at tricellular vertices, as the authors demonstrate using genetic mosaic analysis. The distribution of Sdk strings at vertices changes when junctions shrink during cell intercalation events, suggesting that Sdk is involved in this process. 

Surprisingly, sdk function is dispensable for viability. However, the authors demonstrate that sdk is required for cell intercalation during germband elongation in the Drosophila embryo. Careful quantitative analyses of live imaging data revealed that adhesion at vertices between intercalating cells is compromised in sdk mutants, leading to abnormal cell shapes and persisting apical indentations between intercalating cells. Interestingly, loss of sdk affects cell behavior in an anisotropic fashion, with differential effects on anterior-posteriorly (AP) and dorsoventrally (DV) oriented junctions, respectively, resulting in DV shortening and AP elongation of cells during germband elongation. The authors also analyzed the behavior of Sdk in rearranging multicellular rosette structures, which have previously received considerable attention in the field. They show that rosette centers consist of multiple Sdk puncta, arguing that rosettes in fact contain separable apical vertices
and that intercalation events within these structures reflect multiple T1 transitions. Together, these new findings represent a significant advance for understanding the dynamic behavior of cells during tissue morphogenesis. 

Finally, the authors employed mathematical modeling to support their experimental findings. The computer simulations (the mathematical basis and validity of which this reviewer is unable to assess) reproduced the cell behaviors observed in sdk mutants, supporting the conclusion that a delay in T1 transitions and altered mechanical properties of the rearranging tissue can explain the observed abnormal cell behavior in sdk mutants. 

Overall, this an excellent piece of work with convincing microscopy data that are carefully quantified and appropriately interpreted. The characterization of the structure and function of apical cell vertices provides an important conceptual advance for the field, with significant implications for epithelial biology in many different contexts and model systems. The work should therefore be of interest to a broad audience of readers. 

Two very recent papers in Developmental Cell describe roles of Sidekick in epithelial morphogenesis and have reached conclusions that are largely similar and complementary to those of Finegan et al. (Letizia et al. (2019). Sidekick Is a Key Component of Tricellular Adherens Junctions that Acts to Resolve Cell Rearrangements. Dev Cell 1–33; 
Uechi and Kuranaga (2019). The Tricellular Junction Protein Sidekick Regulates Vertex Dynamics to Promote Bicellular Junction Extension. Dev Cell 1–27). 
Of note, the work by Finegan et al. is complementary to these two papers, as it provides a more in-depth quantitative analysis of cell intercalation in sdk mutants, and it combines the experimental data with mathematical modeling. 

The authors should address the following (minor) points before the work should be accepted for publication: 

The manuscript needs to be carefully checked for typographical errors. 

Fig. 1A: “G6” should presumably read “M6”*
Now corrected.

*Line 197: There is no cartoon in Fig. 2A (but in Fig. 2B).* 
Now corrected.

*Line 88: change “domain” to “motif”*

Now corrected.

*line 442: change “intracellular PDZ domain” to “intracellular PDZ domain-binding motif”*

Now corrected.

*Fig. 2C-E: To support the authors' interpretations, DV as well as AP oriented junctions should be shown for each stage.*

The quantifications in Fig. 2F-H is the basis for our interpretation that the strings are becoming planar during junctional shrinking. In Figure 2C-E, our intention was only to illustrate this interpretation. Because the demonstration is in the quantifications and the images are examples to illustrate our interpretation, we feel it is clearer to not show in addition examples of other orientations (which can be seen in larger field of views, Figure 1B and Figure S1F). To address the referee’s point, we have rewritten extensively this section of the results (lines 231-257) to highlight better the quantifications in Fig. 2F-H and clarified that Fig. 2 C-E is an inference based on the quantifications. We have also rotated the example images in C and D to reflect their orientation in the tissue and annotated the strings with their classification as “planar”, “step” and “vertical”, to link it more clearly to the quantification in panel F.

*Line 152: The colocalisation between Sdk-YFP (extracellular) and anti-Sdk (intracellular) staining (Fig. S1) does not strictly rule out the possibility that Sdk protein may be cleaved, because potential cleavage fragments may remain associated. This does not affect the author´s conclusion that Sdk strings are likely contain the entire Sdk protein (Fig. S1F,G), but the wording should be adjusted accordingly.*

This has now been rephrased (lines 176-178).

*Apical “holes” (Fig. 4A): Holes are not evident from the images shown. Can these be shown more clearly using a membrane marker to outline cell surfaces? Also, a cross-sectional view should be shown to better visualize the “holes”. The term “hole” suggests that a cell or the tissue is perforated, which does not appear to be the case here. The term apical “indentation” therefore appears more appropriate.*

In response to the referee’s comment, we have tried to image the apical gaps in the *sdk*[M5054] mutant labelled with either Gap43-cherry or Spider-GFP, but in absence of a E-Cadherin or a MyoII marker in the same stock, we haven’t managed to generate some convincing images. One problem is that the configuration of membranes can change within the first 2-3 microns, complicating the imaging (see Fig S3). Ideally, we would use a membrane marker and Myosin II in a *sdk*[MB5054] mutant to address this point by SIM, but the OMX microscope we had access to has since been dismantled. What is clearest in our existing data, it that the gaps we find are bordered by Myosin II (Fig. 5A). These Myosin II rings or ovals are usually associated with low Cadherin, which make them hard to visualize in cross-sectional views. In the example shown in Fig. 5B and Figure S6A, there is more Cadherin around the apical gap than usual, so we used this rare example to follow the gap a couple of microns below the surface (Figure 5B). This example shows that the gap is present only in the first micron and is closed below. Based on examples like this, this suggests that the membranes along with the cortex have separated at the level of the adherens junctions, but we cannot be certain with the data we currently have. To help clarify what we mean by apical gaps, we are showing additional examples this time with super-resolution (SIM) in new panels C in Figure 5. We have also adjusted the text throughout the manuscript to remove “holes” and refer them now as gaps or tears in the apical cortex.

*Line 28, 168: The statement that pair-wise homophilic adhesion is sufficient to enrich Sdk at tricellular vertices is misleading, because the term “sufficient” suggests that bicellular homophilic adhesion alone could provide a mechanism for vertex-specific localization of the protein. The genetic mosaic experiment indicates that two (rather than three) cells contributing Sdk are sufficient for localizing the protein at vertices. However, this does not imply that homophilic adhesion alone mediates vertex localization. Additional mechanisms are likely to be involved. The statement should be rephrased.*

We agree and have rephrased this (lines 195-196). Note that different possible mechanisms for vertex localisation are mentioned in the discussion.

*The authors speculate that a sizing mechanism may localize Sdk protein at vertices by excluding it from bicellular spaces and concentrating it at tricellular spaces. They suggest that cell vertices might have larger intercellular spaces than bicellular contacts: This should be readily evident from TEM images. Can the authors show an example to support their hypothesis, or cite a study showing this?*
Rather than a specific study, this idea is based on the theoretical geometry of a vertex with 3 apposed cortices at 120 degrees of each other, which would create a bigger gap at the tricellular vertex compared to the gap between bicellular membranes. Of course, *in vivo*, things could be very different, which is why it would be interesting to investigate. In term of references, in 1982, E. Fristrom (DOI: 10.1083/jcb.94.1.77) has imaged tricellular gaps in *Drosophila* imaginal discs (for example, see Fig. 7 in that paper), but this was at the level of septate junctions, not adherens junctions.

*The authors should comment on whether vertebrate homologues of Sdk also localize at cell vertices.*
To our knowledge, a localisation of Sdk at vertices has not been reported yet- most of the research done in vertebrates has been performed on the neuronal function of Sdk 1 and 2. We now mention this point in the Introduction (line 94-95).

*Reviewer #2: 

Advance Summary and Potential Significance to Field:

In this manuscript, Finegan, Hervieux et al. study the role of the Ig-containing molecule Sidekick (Sdk) which is specifically localised at tricellular junctions at the level of AJs, in the control of cell shape changes and cell rearrangements during gastrulation movement in early Drosophila embryos. This manuscript extends a previous study made by this team reporting the localisation of Sdk at tricellular junctions, bringing better resolution using super-resolution microscopy approaches, and describes defects in ectodermal cells D/V extension and vertices resolutions during germ band extension (GBE) associated with sdk mutations using elaborate quantitative approaches in live embryos. Finally, adopting their previously published mathematical modelling of GBE cell rearrangements they suggest that the observed defects in sdk mutants are for the most part dependent of delayed vertex resolution.

Comments

The main observations of the paper are the fine description of Sdk localisation at tri-cellular junctions and the identification and fine quantification of a sdk mutant phenotype in the embryonic ectodermal cells where sdk mutations result in defects in cell shape and in vertices and rosette resolution during gastrulation movements. While this observation is interesting, the current study remains however very descriptive and lacks molecular insights to start understanding how Sdk at the tricellular adherens junctions could regulate these morphogenetic events. Below are detailed the main points that would need to be addressed before I can support publication.


1. The phenotype associated with sdk loss-of-function is quantified in great details. However, it is rather weak, since the sdk null mutants are homozygous viable and one could question the specificity of the effects observed which might be unrelated to the sdk gene function, and merely reflect genetic background variations. The authors should thus either provide rescue experiments or at least analyse another independent mutation of sdk, such as sdk∆15, and report similar observations.*

When we started this study, only one allele of sdk was available, Sdk[MB5054]. The original allele sdk[P1] described by Nguyen et al 1997 had been lost. We had a personal communication from Jessica Treisman that Sdk[MB5054] was a null allele, based on their molecular studies, which were published recently alongside the characterisation of a new allele sdk[delta15], which is a small deletion (Astigarraga et al., 2018). The movies we analysed in our manuscript had been acquired and segmented with manual correction before a second allele of *sdk* was available. This is a significant amount of work, which we haven’t repeated with the new allele. We had some evidence that the phenotype we analysed is linked to the *sdk* locus: embryos *sdk*[MB5054] over a small deficiency, Df(1)ED6443, show the same cell shape phenotype as *sdk*[MB5054] (Tara Finegan, PhD thesis, 2018). Also, in the course of this work, several stocks and recombinants have been generated (for example recombinants between *sdk*[MB5054] and *sqh*[AX3] on the X chromosome), and all retained the cell shape phenotypes associated with *sdk*[MB5054]. This is of course not a full demonstration, and we agree with the referee that ideally, we would like to repeat our quantifications with sdk[delta15]. This will require making new stocks to combine *sdk*[MB5054] with DE-Cad-GFP, acquiring movies, segmenting and synchronising them, which is beyond the time allocated for this revision. In an attempt to address the reviewer’s concerns, we have immunostained yw, *sdk*[delta7], *sdk*[delta15] and *sdk*[MB5054] embryos with pTyr, to label the adherens junctions and PH3, to mark cell divisions. Sdk[delta7] is a precise excision of the minos element causing the *sdk*[MB505] lesion and should be as wild-type (Astigarraga et al., 2018). PH3 was used to stage the embryos and take images when the midline starts dividing. The cell shape phenotypes are subtle and more difficult to identify in still images than in movies, so to avoid bias we gave 2 independent batches of images of each 4 genotypes to assess blind by a lab member and the first author (who is now in the US). Both identified correctly *sdk*[delta15] as having a *sdk*-like phenotype and *sdk*[delta7] as having a WT-like phenotype. An example of each genotype is given below (the PH3 channel is not shown, the midline is at the top of each image and the cephalic furrow on the left). Note that two other studies on *sdk* uses the *sdk*[MB5054] allele and provide genetic evidence that the cell rearrangements defects they observe are linked to the *sdk* locus (Letizia et al 2019; Uechi & Kuranaga, 2019).

*As a note here, since sdk null mutants are viable, the defects observed should remain transient and be resolved later in development. Are the differences observed in Figure 3D and 3H on axial shape elongation and DV interface length ever resolved or is the embryo making do with misshapen ectodermal cells?*

We think that the cell shape phenotypes that remain at the end of germ-band extension are resolved by cell division. This is mentioned in the main text (now line 441) and an example is shown in Figure 6B: the dividing cells cause local cell rearrangments which resolve a stuck rosette. Since all the cells in the ectoderm eventually divide, we propose that this is how normal apical cell shapes are restored in *sdk* mutants. In response to the reviewer, we now mention this point in the discussion (line 867).

*2. With respect to the sdk mutant phenotype, the observation of the occurrence of holes/gaps in the E-Cadherin and junctions that last longer than in wild-type, is really interesting. Can this phenotype be modified, for instance by lowering the dosage of the shg or arm genes? This would bring more weight to the specificity of the phenotypes reported.*

We agree that lowering the dosage of arm/shg would be good experiments, but we feel this is beyond the scope of this paper. Note that in the revised manuscript, we now provide super-resolution images of the gaps in the cortex in new panel C in Figure 5.

*Authors report this defect in particular at the level of rosettes. Even though not explicitly stated, one could thus anticipate that rosettes accumulate over time as gaps at vertices fail to resolve. Authors should thus quantify the number of rosettes over time in wild-type and sdk mutants.*

The referee’s prediction is in agreement with what our simulations show: in the WT simulation, the rosettes appear and disappear in the course of extension, while in the *sdk* simulations, rosettes appear and accumulate until the end of extension (interestingly, more rosettes appear in the second simulation, where the mechanical properties of the tissue have been changed). We would love to quantify rosettes over time in wild-type and *sdk* mutants, however we cannot do this at present with the tracking framework we are using, because the tracking records each cell-cell interfaces as “immortal” throughout a movie. Method development for this would take time which is beyond what is possible for this revision. Note that our quantification of gap persistence at rosettes gives indirectly information on rosettes numbers in WT and *sdk* mutants. In response to the reviewer point, we have now highlighted rosettes in the simulations, in Figure 7 and in 3 movies of the simulations now provided.

*A side note of Figure 4; authors state that holes last longer in sdk mutants, but authors should provide the reader with the information about the relative length of the GBE process in both wild-type and sdk mutants. If the whole development and GBE are slower in sdk mutants, some of the interpretations on persistence of this particular features might have to include that the whole development is slower, and not just the vertices resolution.*

We haven’t considered this since Nomarski imaging of sdk[MB5054] compared to wildtype did not reveal any developmental delays (Tara Finegan, PhD thesis, 2018). As a control for this, the onset of cell divisions for each movie tracked in our paper is presented below. Although the timings of the first divisions either at the midline (left graph) or in the dorsal ectoderm (right graph) are highly variable, this quantification suggests that there is no significant difference between WT and *sdk* development (if anything, *sdk* mutants might be a little ahead).

*3. The current study fails to bring any hints at the molecular mechanisms underlying the sdk mutant phenotype. Given the importance of Myosin planar orientation for some of the features measured here such as DV vs AP axis of junction elongation/shrinkage or rosette formation, and given the importance of Bazooka and of the E-Cadherin complexes for AJ remodelling, authors should study what are the effects of sdk mutations on MyosinII, Bazooka, E-Cad, and alpha- and beta-Catenin distribution either on live or fixed embryos. This is especially important with respect to the E-Cad holes observed in sdk mutants.*

We agree that these analyses will be important but we feel that these are beyond the scope of this paper. The work we present in this paper was aimed at characterising the whole tissue behaviour during germ-band extension rather than the cell biology. To study the distribution of these markers adequately, we will need to work at a different scale and acquire movies with higher spatial and temporal resolution. As mentioned above, we have added some super-resolution images of the gaps we observe in *sdk[5054]* mutants, which shows more precisely the distribution of E-Cadherin and Myosin II at these structures (Figure 5C). Note that with the exception of these gaps, we haven’t noticed any obvious defects in Myosin II and E-cadherin distribution in *sdk* versus WT in our images of fixed or live embryos.

*4. Finally, while mathematical modelling is provided here, it is not clear how close the different tweaks added and the mathematical model predictions match the observations. Authors should thus show how the different parameters measured in Figure 3 (or at least some of them) and which appear grossly independent from the “delayed vertices resolution” constant that has been modified in the model, are changed and whether they match the experimental data. Authors could also indicate whether the model predicts the perdurance of rosettes as seems to be observed in the experiments (see point 2).*

We have expanded this part in several ways in the revised manuscript: i) we are now highlighting rosettes in the simulations, as mentioned in response to point 2, ii) we have tested the prediction of a delayed resolution in T1 swaps in the real data. This analysis shows that there is a measurable delay between WT and *sd*k[MB5054] mutants, of 1 minute (new panels B and C in Figure S9). While this is a small difference, this measurement excludes stuck rosettes because only successful T1 transitions are considered in the analysis. So we conclude that successful T1 transitions, most likely in tetrads, are delayed in *sdk* mutants. This justifies the lower probability given to tetrad resolution in the *sdk* simulations. Note that delays in resolution have also been observed in a new paper on *sdk* in the genitalia disc (Uechi & Kuranaga, 2019).

*More minor points

A. Authors should try to link more clearly, at least in their interpretations/discussions, the dynamics of Sdk during D/V oriented boundary shrinkage (Figure2) and the phenotypes observed in Figure 3/4/5.*

It is difficult without over-interpreting since we don’t know if the apparent localisation of Sdk at shortening junctions is because of thin tricellular protrusions or presence at the bicellular shortening contact (see Fig. S1 H,I). Future studies will need to resolve this first before we can propose some mechanisms.

*--

Reviewer #3 (Mark Peifer): 

One key question for the cell and developmental biology field is to define mechanisms that allow cells to change shape and move, which requires force generation and major remodeling of cell-cell junctions, without disrupting tissue integrity.  Several recent studies have suggested that the actomyosin force-generating structures may be anchored to the cadherin-based cell adhesion machinery at tricellular and multicellular junctions.  The authors (and others) have identified a protein, Sidekick, that is highly concentrated at tricellular and multicellular adherens junctions.  They combine super-resolution microscopy, genetic analysis, and powerful quantitative and modeling tools to explore the structure of tricellular junctions and the function of Sidekick.  I found the results both compelling and exciting.  I have some suggestions for a modest set of experiments that would amplify on what is present as well as some suggestions for clarification.  This will be of broad
interest to cell and developmental biologists.

Suggested experiment
Several proteins have been found that concentrate at tricellular junctions during the stage the authors are studying (though not as completely as Sidekick), and which have known or predicted roles in the process.  It would be quite interesting to see super-resolution images of tricellular junctions during germband extension double labeled with Sidekick and non-muscle myosin, Canoe and Enabled.*

We agree that these are good experiments but we feel this is beyond the scope of this revision. As mentioned in response to referee 2 point 3, the work we present in this paper was aimed at characterising the whole tissue behaviour during germ-band extension rather than the cell biology.

*Data questions and clarifications
The combination of experiments, quantitative analysis and modeling is extremely powerful.  However, several of the approaches used are described quite briefly and thus were hard for a simple-minded cell biologist like me to follow.  Slight amplifications would allow us to appreciate what was being measured.
1.	 Fig 5 and associated text.  Add a more complete description of the different types of strain rates to go with the nice diagrams.*

We have now added more text to explain the strain rate measurements (lines 501-507).

*2.	 P. 10—this goes through an extremely complex series of measures of intercalation—mostly supplemental.  Perhaps focusing in the main text on those which were the most informative?*

In response, we have removed our Voronoi analysis (see below) and edited the text. The main text now focuses on two methods for measuring polarised cell intercalation, a continuous method based on strain rates, and a discrete method counting the T1 swaps.

*3.	p. 11 what is a Voronoi tiling*

This is a mathematical method to partition a plane into regions or cells based on seed points. We have used Voronoi tiling in the past based on known cell centroid locations as seed points to compare the configuration of Voronoi cells with the configuration of real cells in the tissue (Tetley et al 2016). Sdk and WT deviate in distinct ways from Voronoi tiling. Because this measure is about cell geometry and some assumptions are required to link this to tension differences during polarised cell intercalation, we decided to remove it to simplify the text.

*The simulations in Figure 6 are a central part of the paper but I felt the authors did not explain them very clearly.  My interpretation of these is that the 1st simulation does not match observed tissue strain rates—they should explicitly tell us this and then use the 2nd simulation to add what they now view as an important parameter and walk us through how this improves the match.  As part of this they should annotate their resulting cell shape outputs:  for example, it’s not obvious that 6G is that different from 6E—point out places where it is and then use additional arrows to point out how 6I is much more similar to what is observed in the mutant*

We have edited the text to more explicitly describe the two different *sdk* simulations. To make the cell shape changes more apparent, we have increased the size and resolution of the corresponding figure (now Figure 7, panels D-I). We are now also highlighting the rosettes in the simulations and include a movie per simulation (movies S3 to S5). *There was one place where I thought there was an overstatement of facts I think needs to be corrected.  In both the results and the Discussion the authors make a claim I think needs to be tempered—an example is Discussion-p. 13  “Sidekick, in contrast, is present only at tricellular contacts”.  When I examine their superresolution imaging (e.g., Fig. 1D,E) it seems like this is simply not true.  It’s highly concentrated there, but there is signal along bicellular borders.  This should be toned down.*

We have removed “only” and also expanded a paragraph in the discussion about the cases where Sdk is found outside tricellular junctions (lines 778 onwards). The reason for the strong statement was that we wanted to make clear that Sdk localisation is very different from the localisation of other proteins that are bicellular and also enriched at vertices. This is based on the work we did when we found the vertex localisation of Sdk, while screening 600 YFP-tagged CPTI lines by visual inspection in the early embryo (Lye et al 2014). Here is what we wrote on Sdk localisation in this paper: “Sdk-YFP initially localises as spots at stage 5 that appear more concentrated in the vicinity of tricellular vertices and have not all reached an apical position (Fig. 5A,B). At stage 8 and beyond, Sdk-YFP becomes fully apical and is also mostly excluded from bicellular contacts (Fig. 5C). This is confirmed by a co-staining with p-Tyr (Fig. 5D,D′). Although Sidekick is the only protein found in the screen whose membranous localisation is mostly at vertices, we found a large number of membranous/cortical proteins that are enriched there at stages 6 to 10 (30 lines inserted in 24 genes, supplementary material Table S4). The majority are apically localised and include Canoe, whose enrichment at vertices was previously reported in early embryos (Sawyer et al., 2009). Consistent with the vertex enrichment of actin also reported in that study, most proteins localising at tricellular vertices in our screen are actin-binding proteins or actomyosin regulators (supplementary material Table S4).”

*Introduction
There are a number of places in the Drosophila and mammalian literature where people have explored tension at tricellular and multicellular junctions and proteins that are enriched there.  A slightly expanded section on this in the Introduction would help put this work in context.*

We intentionally didn’t expand on tension in the introduction because we do not address this directly in our manuscript and prefer to keep the introduction brief. We have edited the sentence and added a couple of references relevant to tension at tricellular vertices.

*Tell us directly in the Introduction that Sidekick loss is non-essential.*

To address this comment we have now written a paragraph in the discussion (rather than in the introduction) about the viability of Sdk and what it could mean in term of redundant mechanisms during epithelial morphogenesis. We also mention the two articles on Sdk recently published in this last section. *Minor points
p. 5, paragraph 1.  Reference figure panels—is it all in S2?  Several things in Fig, S1 and S2 could use explanatory arrows (e.g lines 150-152, 167-169*

Our survey of the localisation of Sdk in diverse tissues is presented exhaustively in Table 1, with some illustrative examples in Figure S2.  *p. 5, paragraph 2.  I found it hard to visualize the directionality of the “strings”—X/Y versus Z.  Walk us through this a bit more clearly.*  
We have edited the text in response.

*p. 7, lines 186 ff.  Rephrase “In this data, Sdk signal along DV-oriented junctions….”*

This section has now been rewritten.
 *The data in Fig S3A-D is quite interesting and might be moved to a main figure.*

We agree and this is now Figure 3.  *PS  I loved this idea and saw an intriguing talk at a Gordon Conference this summer that tested the basic mechanism in a simplified model—I wonder if there is an existing literature that might be cited for the underlying principle?  “Because of their geometry, vertices in epithelia might have larger intercellular spaces than bicellular contacts. A possibility therefore is that Sdk resides at tricellular contacts because of a sizing mechanism that excludes Sdk from bicellular spaces and concentrate it at tricellular spaces”*
See response to similar point by reviewer 1.
